# The Cambridge Centre for Ageing and Neuroscience (Cam-CAN) data repository: Structural and functional MRI, MEG, and cognitive data from a cross-sectional adult lifespan sample

**DOI:** 10.1016/j.neuroimage.2015.09.018

**Published:** 2017-01

**Authors:** Jason R. Taylor, Nitin Williams, Rhodri Cusack, Tibor Auer, Meredith A. Shafto, Marie Dixon, Lorraine K. Tyler, Cam-CAN, Richard N. Henson

**Affiliations:** a School of Psychological Sciences, The University of Manchester, Zochonis Building, Brunswick Street, Manchester M13 9PL, UK; b MRC Cognition and Brain Sciences Unit, 15 Chaucer Road, Cambridge CB2 7EF, UK; c Brain and Mind Institute, University of Western Ontario, London, ON, Canada; d Department of Psychology, University of Cambridge, Cambridge CB2 3EB, UK; e Cambridge Centre for Ageing and Neuroscience (Cam-CAN), University of Cambridge and MRC Cognition and Brain Sciences Unit, Cambridge, UK

**Keywords:** ACE-R, Addenbrooke's Cognitive Exam, BOLD, blood-oxygenation level-dependent, Cam-CAN, Cambridge Centre for Ageing and Neuroscience, DARTEL, diffeomorphic anatomical registration through exponentiated lie algebra, DWI, diffusion-weighted imaging, DTI, diffusion tensor imaging, DKI, diffusion kurtosis imaging, ERF, event-related field, fMRI, [functional] magnetic resonance imaging, GM, grey matter, MNI, Montreal Neurological Institute, MT, magnetisation transfer, MEG, magnetoencephalography, MMSE, Mini Mental Status Exam, ROI, region of interest, VBM, voxel-based morphometry, WMS-III UK, Wechsler Memory Scale Third UK Edition, WM, white matter, Data repository, Brain imaging, Magnetic resonance imaging, Magnetoencephalography, Cognition, Ageing

## Abstract

This paper describes the data repository for the Cambridge Centre for Ageing and Neuroscience (Cam-CAN) initial study cohort. The Cam-CAN Stage 2 repository contains multi-modal (MRI, MEG, and cognitive-behavioural) data from a large (approximately N = 700), cross-sectional adult lifespan (18–87 years old) population-based sample. The study is designed to characterise age-related changes in cognition and brain structure and function, and to uncover the neurocognitive mechanisms that support healthy cognitive ageing. The database contains raw and preprocessed structural MRI, functional MRI (active tasks and resting state), and MEG data (active tasks and resting state), as well as derived scores from cognitive behavioural experiments spanning five broad domains (attention, emotion, action, language, and memory), and demographic and neuropsychological data. The dataset thus provides a depth of neurocognitive phenotyping that is currently unparalleled, enabling integrative analyses of age-related changes in brain structure, brain function, and cognition, and providing a testbed for novel analyses of multi-modal neuroimaging data.

## Highlights

• Cross-sectional uniform adult-lifespan population-based data
• Multimodal MRI, fMRI and MEG neuroimaging data
• Unprecedented depth of cognitive phenotyping
• Age-related differences in brain structure, function, and cognition

## 1 Cam-CAN project

### 1.1 Overview

The Cambridge Centre for Ageing and Neuroscience (Cam-CAN) Stage 2 cohort study is a large-scale (approx. N = 700), multi-modal (MRI, MEG, and behavioural), cross-sectional, population-based adult lifespan (18–87 years old) investigation of the neural underpinnings of successful cognitive ageing. The project is an interdisciplinary collaboration involving researchers with expertise in cognitive psychology, cognitive neuroscience, psychiatry, engineering, and public health. The full Cam-CAN study consists of three stages, described briefly below (for full protocol, see Shafto et al., 2014). The focus of the present paper is the Stage 2 dataset, which includes raw and preprocessed MRI, MEG, and cognitive-behavioural data, along with demographic data and other cross-referenced measures collected from the Stage 2 cohort in Stage 1. Collection of Stage 3 data has recently completed, and these data will be added to the database in due course.

A key focus of the Cam-CAN project is integrative analysis across domains of cognition and measures of neural structure, function, and connectivity, with the goal of understanding how neurocognitive systems adapt in order to overcome age-related changes. For example, Tsvetanov et al. (2015) combined T2*-weighted resting-state fMRI data with resting-state MEG data to show that ageing affects the vascular response independently of neural activity; Geerligs et al. (in press) showed how age-related differences in functional connectivity change with cognitive state; and Kievit et al. (2014) combined fractional-anisotropy measures from the diffusion-weighted (DWI) MRI data with volumetric measures from the T1-weighted MRI data to show that white matter (WM) and grey matter (GM) in frontal cortex make independent contributions to age-related declines in fluid intelligence and multitasking.

Several features of the Cam-CAN Stage 2 dataset together make it unique. First, the sample is derived from a larger, population-based sample (approximate N = 3000) recruited from the general population via Primary Care Trust lists, which can be related to national data (Shafto et al., 2014), and which allows quantification of bias in the Stage 2 sample of people willing and able to undergo neuroimaging. Second, the distribution of ages was selected to be roughly uniform, allowing sufficient statistical power to test for differences within as well as across age groups. Third, Stage 2 of the study involved collecting a broad range of behavioural measures from 14 experiments spanning five main cognitive domains (attention, emotion, action, language, and memory), which can also be related to considerable amounts of demographic, health and lifestyle data obtained in Stage 1. Finally, Stage 2 includes a wide range of neuroimaging measures: high-resolution (1 mm^3^) T1- and T2-weighted images, diffusion-weighted images (DWI), magnetisation-transfer (MT) images, and BOLD EPI images during rest, a sensorimotor task and movie-watching, as well as MEG data during rest and the same sensorimotor task. The depth of this neurocognitive phenotyping is currently unparalleled, and provides a testing ground for the development of new multimodal analysis methods.

In the following section, we briefly describe the three Cam-CAN data-collection stages. Acquisition and analysis of the MRI and MEG data in Stage 2 are described more fully in Section 2.

### 1.2 Data-collection stages

#### 1.2.1 Stage 1

In Stage 1^1^, 2681 participants were interviewed in their homes to acquire demographic information; measures of cognitive, mental and physical health; and lifestyle information. Tests of vision, hearing, balance, and speeded response times were administered, and participants completed detailed self-report questionnaires about their physical activity and life experiences. Neuropsychological tests included general cognitive assessments (MMSE, Folstein et al., 1975; ACE-R, Mioshi et al., 2006a, Mioshi et al., 2006b), tests of memory (logical memory from WMS-III UK, Wechsler, 1999), and verbal intelligence (Spot the Word, Baddeley et al., 1993).

Measures taken in Stage 1 additionally served to screen participants for participation in Stage 2: To continue, participants were required to be willing to continue, be cognitively healthy (MMSE > 24), to meet hearing, vision, and English language ability criteria necessary for completing experimental tasks, and to be free of MRI or MEG contraindications and neurological or serious psychiatric conditions.

#### 1.2.2 Stage 2

In Stage 2, participants (target N = 700: 50 men, 50 women from each age decade) were recruited to attend testing sessions at the Medical Research Council (UK) Cognition and Brain Sciences Unit (MRC-CBSU) in Cambridge, UK. Owing to recruitment problems for the youngest decade (18–27), only 56 (27 men) were tested from this decade. In this stage, structural and functional MRI scans, MEG recordings, and cognitive task data were collected over three separate sessions. Structural and functional MRI scans collected in Stage 2 are listed in Table 1, Table 2; MEG recordings are listed in Table 3; and cognitive behavioural tasks are listed in Table 4. Physiological data (height, weight, and blood pressure) were also collected, and a saliva sample was taken for future genetic analysis. As the data from Stage 2 are the focus of the repository described in this paper, these data and derived measures are described in more detail in Section 2.

#### 1.2.3 Stage 3

In Stage 3, a subset of participants (target N = 280: 20 men, 20 women from each decade) were recruited to attend further MRI and MEG sessions within 3 years of their assessment in Stage 2. Over three sessions, structural MRI and physiological measures were collected, along with fMRI and MEG data on a variety of cognitive tasks. Structural MRI scans (all participants) included a repeat T1-weighted structural image, as well as T2-weighted FLAIR, and arterial spin labelling (ASL). Functional MRI tasks (target N = 140 each) investigated emotion regulation, emotional memory, fluid intelligence, picture naming, response selection and inhibition, sentence comprehension, and visual short-term memory; repeat resting-state data and field maps for distortion correction were also collected. Height, weight, and blood pressure, which were measured in Stage 2, were re-measured at the Stage 3 MRI session. MEG tasks investigated incidental memory, oddball processing, picture naming, response selection and inhibition, sentence comprehension, and word recognition, as well as a resting state. Hearing, vision, and cognitive status (MMSE), which were also measured in Stage 1, were re-evaluated in the MEG session.

## 2 Database details

### 2.1 Purpose

Using semi-automated Matlab and Linux shell scripts, raw data were pulled from various sources (testing laptops, MRI and MEG data servers) into a central location. Once there, further automated scripts identified new raw data and submitted them to the appropriate processing scripts. Behavioural data were analysed by custom Matlab scripts; MRI and MEG data were processed using Automatic Analysis (aa 4.2; Cusack et al., 2014) pipelines and modules which called relevant functions from neuroimaging analysis software and toolboxes (SPM12, Wellcome Department of Imaging Neuroscience, London, UK; FSL, Smith et al., 2004; Freesurfer, Martinos Center for Biomedical Imaging, Massachusetts General Hospital, Boston, MA, USA; in-house code).

The remit of the database is limited to the Cam-CAN initial study cohort project. Therefore, upon completion of data collection and preprocessing analyses, the repository will effectively be static (i.e., no further raw data will be added). However, as existing pipelines are improved, new data releases are made, and as new pipelines are developed, new processed data will become available. Further, any future studies that use the same cohort and adopt a similar data-sharing policy will likely be incorporated into the database (e.g., there are plans to re-test the Stage 2 cohort on a subset of the same cognitive and neuroimaging measures in the future, to provide longitudinal data).

### 2.2 Contents

The Stage 2 repository contains MRI, MEG, and behavioural data from 656 participants aged 18–87 years old. All data are labelled with unique project IDs. Data were quality-control checked by semi-automated scripts monitored by the Cam-CAN methods team. All analysis scripts (including aa modules and recipes) are stored in the repository and can therefore be viewed by any user. Matlab scripts are also available to query the repository and compile data cross-referenced by participant identifiers.

#### 2.2.1 MRI

##### 2.2.1.1 MRI data collection

All MRI datasets were collected at a single site (MRC-CBSU) using a 3 T Siemens TIM Trio scanner with a 32-channel head coil. Participants were scanned in a single 1-hour session. Before scanning, physiological measurements were taken, and two behavioural experiments were run. In the scanner, memory foam cushions were used for comfort and to minimise head movement. Instructions and visual stimuli for functional tasks were back-projected onto a screen viewed through a mirror mounted on the head coil; auditory stimuli were presented via MR-compatible etymotics headphones; and manual responses were made with the right hand using a custom-built MR-compatible button-box. Cardiac data were recorded using photoplethysmograph/pulse-oximeter on the left index finger, sampled at 50 Hz.

MRI data collected were structural (T1, T2, DWI, and MTI) and functional (resting-state, movie-watching, and sensorimotor task); details are provided in Table 1, Table 2. For the resting state scan, participants rested with their eyes closed for 8 min and 40 s. In the movie-watching task, participants watched and listened to an excerpt of a compelling but unfamiliar film: Alfred Hitchcock's “Bang! You're Dead”, a black-and-white television drama. The film was edited from its original running time of 30 min down to 8 min while maintaining the essential plot (Hasson et al., 2010). Finally, in the sensorimotor task, also 8 min and 40 s long, participants detected the presentation of two circular checkerboards visually presented simultaneously to the left and right of a central fixation cross (34 ms duration) and a binaural tone (300, 600, or 1200 Hz; equal numbers of trials pseudorandomly ordered; 300 ms duration), presented either simultaneously (120 trials + 1 initial practice trial), or separately (8 trials; 4 visual only, 4 auditory only; included to discourage strategic responding to one modality). These task trials were combined with null trials of the same length, and all trials were pseudorandomly ordered using a 255-length m-sequence (m = 2 and minimal SOA of 2 s; Buracas and Boynton, 2002), resulting in effective stimulus onset asynchronies (SOAs) ranging from 2 to 26 s. Participants responded by pressing a button with their right index finger whenever they saw or heard any stimuli.

All MRI data are available in standard NIfTI-1.1 format using single file (.nii) storage (3D for structural and 4D for functional); derived measures (e.g., ROI data) are available in ASCII text or Matlab data formats. All MRI analysis was conducted in SPM12, automated and parallelised by aa, except where noted.

##### 2.2.1.2 MRI data analysis

**Fig. 1:**
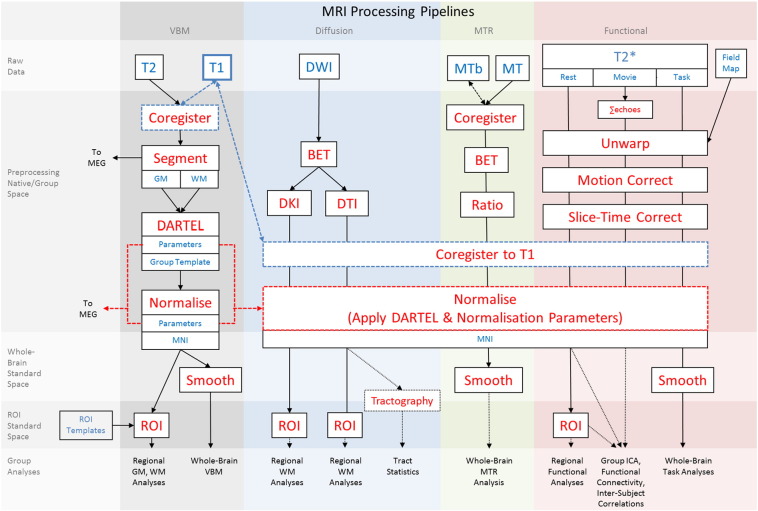
Schematic illustration of MRI processing pipelines. Coloured columns indicate processing stream (see corresponding labels); shaded rows indicate stage of processing (see corresponding labels). Blue text indicates a data type; red text indicates a processing step; dashed lines and boxes emphasise important and unique steps in the pipelines (coregistration of all images to T1; normalisation to MNI by applying flow field parameters computed during DARTEL processing); dotted lines and boxes illustrate planned analyses. See text for a complete description. Notes: Abbreviations as in footnote 1 and text; Mb = magnetisation transfer baseline; ∑ indicates weighted sum.

We describe the main processing pipelines developed and applied to date, but emphasize that other pipelines are possible and may be superior. MRI data were processed in separate streams (see Fig. 1): (i) a voxel-based morphometry (VBM) stream for structural analysis; (ii) a DWI stream with separate branches for diffusion tensor imaging (DTI) with nonlinear fitting (Correia et al., 2009) and diffusion kurtosis imaging (DKI) (Henriquesa et al., 2015); (iii) an MTR stream; and (iv) an fMRI stream with separate branches for resting state, movie-watching, and sensorimotor tasks. Each stream was independent of the others, with two exceptions: Image types from each participant were coregistered to that participant's T1-weighted image via a rigid-body (6-df) linear transformation (blue dashed lines in Fig. 1), and normalisation parameters from the DARTEL procedure in the VBM stream were applied in the normalisation stages of the other streams (red dashed lines in Fig. 1), as described below. This procedure ensures a voxel-to-voxel correspondence for all metrics derived from each modality (e.g., GM volume, FA, MTR, BOLD timeseries, etc.). Each processing stream was applied to each individual participant's data independently, with the exception of the DARTEL group template stage in the VBM stream and later stages of fMRI processing (group ICA, inter-subject correlations, and 2nd-level analysis of task-related activity). Structural images have been de-faced (Bischoff-Grethe et al., 2007) in order to protect participant anonymity.

In the VBM stream, the T1 image was initially coregistered to the MNI template, and the T2 image was then coregistered to the T1 image using a rigid-body (6-df) linear transformation. The coregistered T1 and T2 images were used in a multi-channel segmentation (SPM12 Segment, based on “New Segment” in SPM8; Ashburner and Friston, 2005) routine in order to extract probabilistic maps of 6 tissue classes: GM, WM, cerebrospinal fluid (CSF), bone, soft tissue, and residual noise. The native-space GM and WM images for all participants who passed quality-control checks (N = 651 in the current release003) were then submitted to diffeomorphic registration (DARTEL; Ashburner, 2007) to create group template images. The group template was then normalised to the MNI template via an affine transformation, and combined normalisation parameters (native to group template and group template to MNI template) were applied to each individual participant's GM and WM images. From this stage, the structural analysis stream followed two branches: For VBM analysis, individual normalised GM and WM images were smoothed (8 mm FWHM Gaussian kernel); for region of interest (ROI) analysis, applied masks from anatomically labelled template images (Harvard-Oxford atlas, Desikan et al., 2006, Craddock et al., 2012; in separate sub-branches) to extract mean regional GM and WM signal values for each participant.

In the DWI stream, data were first skull-stripped using the BET utility in FSL. From there, two parallel branches were implemented: One for nonlinear estimation of the typical second-order diffusion tensor (and associated derived metrics, such as FA, mean diffusivity (MD), radial and axial diffusivity (RD, AD), etc.), and another for estimation of higher-order moments like kurtosis (and associated derived metrics, such as mean, radial, and axial kurtosis (MK, RK, AK); both DTI and DKI pipelines use in-house code).

In the fMRI stream, the multiple echoes collected in the movie-watching task were first combined. Then, data from each functional run (resting state, movie-watching, and sensorimotor task) were unwarped (using field-map images) to compensate for magnetic field inhomogeneities, realigned to correct for motion, and slice-time corrected. After the EPI data were co-registered to the T1 image, the normalisation parameters from the VBM stream were then applied to warp functional images into MNI space. From this stage, three branches split off: For ROI analysis, mean regional time-courses were extracted using the template method described in VBM above; for task-related fMRI analyses, normalised images were smoothed (12 mm Gaussian kernel), and a general linear model (GLM) was applied with regressors defined by stimulus onsets (simultaneous and unimodal events separately) and 6 motion parameters. Further fMRI analysis pipelines are in development, for example, i) to assess functional connectivity using independent components analysis (ICA), ii) remove residual motion artifacts using wavelet de-spiking (Patel et al., 2014) and regression of WM/CSF signals and higher-order expansions of the movement parameters (Geerligs et al, in press), iii) calculate resting-state fluctuation amplitudes (RSFA) in order to scale task activations (Tsvetanov et al., 2015), and iv) investigate voxel-wise inter-subject correlations evoked by the movie-watching task.

The final number of participants whose MRI data were processed to completion, i.e., have ROI data for T1, T2, DWI, MT and T2* (for rest, movie and sensorimotor task) at end of paths in Fig. 1 (excluding those with artifacts, missing or incomplete data, etc), is 614 (numbers per seven decades from 18 to 87, respectively: 51, 101, 93, 91, 96, 93, 89).

#### 2.2.2 MEG

##### 2.2.2.1 MEG data collection

All MEG datasets were collected at a single site (MRC-CBSU) using a 306-channel VectorView MEG system (Elekta Neuromag, Helsinki), consisting of 102 magnetometers and 204 orthogonal planar gradiometers, located in a light magnetically shielded room (MSR). Data were sampled at 1 kHz with a highpass filter of 0.03 Hz. Recordings were taken in the seated position. Head position within the MEG helmet was estimated continuously using four Head-Position Indicator (HPI) coils to allow for offline correction of head motion. Two pairs of bipolar electrodes were used to record vertical and horizontal electrooculogram (VEOG, HEOG) signals to monitor blinks and eye-movements, and one pair of bipolar electrodes records the electrocardiogram (ECG) signal to monitor pulse-related artefacts. Instructions and visual stimuli were projected onto a screen through an aperture in the front wall of the MSR; auditory stimuli were presented via etymotic tubes; responses were made via a custom-built button box with fibre optic leads.

MEG data were collected during resting state, a sensorimotor task, and an audio-visual (passive) task (see Table 3). During the resting state recording, participants sat still with their eyes closed for at least 8 min and 40 s, to match the fMRI resting state scan. During the sensorimotor task recording, participants performed the same task as in the fMRI version. The audio-visual (passive) task used the same stimuli as the sensorimotor task, but with visual and auditory stimuli presented in isolation rather than simultaneously (in order to facilitate separation of the MEG responses evoked by each modality), and participants were not required to respond. In this task, 120 trials of unimodal stimuli (60 visual bilateral checkerboards presented simultaneously, 60 auditory tones at one of three equiprobable frequencies; see definition of stimuli in MRI section above) were presented at a rate of approximately 1 per second.

Raw and maxfiltered MEG data are available in Neuromag's FIF format; subsequently preprocessed data are available in SPM12 format, with output from some intermediate stages (e.g., ICA) in Matlab format. All MEG analyses were implemented using aa.

##### 2.2.2.2 MEG data analysis

**Fig. 2:**
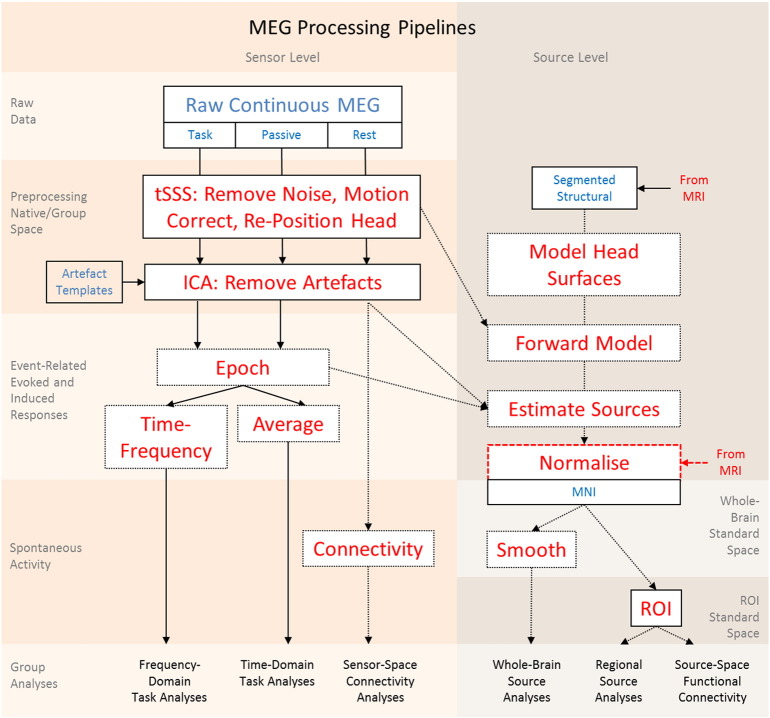
Schematic illustration of MEG processing pipelines. Coloured columns indicate sensor-space and source-space streams (see corresponding labels); shaded rows indicate stage of processing (see corresponding labels). Blue text indicates a data type; red text indicates a processing step; dotted lines and boxes illustrate planned analyses. See text for a complete description. Notes: Abbreviations as in footnote 1 and text; tSSS = temporal extension of signal space separation.

The MEG preprocessing pipeline is illustrated in Fig. 2. For each run, temporal signal space separation (tSSS, Taulu et al., 2005; MaxFilter 2.2, Elekta Neuromag Oy, Helsinki, Finland) was applied to continuous MEG data to remove noise from external sources and from HPI coils (correlation threshold 0.98, 10-sec sliding window), for continuous head-motion correction (in 200-ms time windows), and to virtually transform data to a common head position (‘-trans default’ option with origin adjusted to the optimal device origin, [0,+13,− 6]). MaxFilter was also used to remove mains-frequency noise (50-Hz notch filter) and to automatically detect and virtually reconstruct any noisy channels. Data were then imported to SPM12 format. ICA was used to identify physiological artefacts from blinks, eye-movements, and pulse, i.e. those ICs that had the highest correlation with the VEOG, HEOG and ECG channels respectively. These 3 ICs were then projected out of the data.

For the sensorimotor and passive audio-visual tasks, evoked analysis of event-related fields (ERFs) and time–frequency analysis of induced signals were performed, time-locked to stimuli and manual responses. Further pipelines are being developed for the MEG data, including source estimation of task-evoked signals (using a head model defined using each participant's structural MRI), and connectivity analysis of sensor- and source-level data (e.g., using multivariate autoregressive (MVAR) modelling or correlation of amplitude envelopes).

The final number of participants whose MEG data from all three sessions (rest, sensorimotor task, and passive audio-visual task) were successfully processed to the stage of artefact rejection in Fig. 2 is 623 (numbers per seven decades from 18 to 87, respectively: 47, 102, 100, 97, 94, 93, 90). The final number of participants with complete MRI and complete MEG data is 572 (numbers per seven decades: 44, 96, 90, 86, 89, 84, 83).

#### 2.2.3 Behavioural task data

Behavioural data were collected on 14 cognitive tasks selected to assess five core cognitive domains: executive function, emotional processing, motor and action function, language processing, and memory. Most experiments were computerised tasks run on a laptop computer using E-Prime v1 or v2, Visual Basic, the Cogent toolbox for Matlab; several were paper-and-pencil tasks, with data input via custom Matlab scripts; the remainder required specialist equipment or were naturalistic table-top tasks. A brief description of each task is given in Table 3; for full descriptions, see Shafto et al. (2014). Demographic information is also available from Stage 1. Behavioural and demographic data are available in ASCII (.txt) format.

#### 2.2.4 Physiological measures

Height was measured with a portable stadiometer with a sliding head plate, a base plate and a connecting rod marked with a measuring scale. Weight was measured with a portable battery operated electronic weighing scales. Blood pressure measures were measured with a Digital Blood Pressure Monitor (A&D Medical UA-774); three measurements were taken in order to ensure reliability. These physiological measures are available in the behavioural data section of the repository. A saliva sample was also collected for future genetic analyses.

### 2.3 Access

Currently, the repository is currently stored on a Linux file system and can be accessed via secure shell (SSH) connection or secure file-transfer (SFTP) protocol. Instructions for accessing the data are available here: http://www.mrc-cbu.cam.ac.uk/datasets/camcan/.

Future studies based on the same or similar samples will be incorporated into the database.

## 3 Summary

The Cam-CAN Stage 2 data repository is a large, multi-modal, cross-sectional adult lifespan dataset designed to facilitate characterisation of age-related changes in cognition and brain structure and function, and to enable analysis aimed at discovering neurocognitive mechanisms that support healthy cognitive ageing. The database contains raw and preprocessed structural MRI, functional MRI (active tasks and resting state), and MEG data (active tasks and resting state), derived scores from cognitive behavioural experiments, and demographic and neuropsychological data. This repository of multi-modal neuroimaging and cognitive behavioural data thus provides an unprecedented depth of neurocognitive phenotyping, enabling integrative analyses of age-related changes in brain structure, brain function, and cognition.

## Figures and Tables

**Table 1:** Structural MRI scans collected in Stage 2.

Scan type	Sequence	TR (ms)	TE (ms)	Flip angle (°)	FOV (mm)	Voxel size (mm)	Other
T1-weighted	MPRAGE	2250	2.99	9	256 × 240 × 192	1 × 1 × 1	GRAPPA: 2; TI: 900 ms
T2-weighted	SPACE	2800	408	9	256 × 256 × 192	1 × 1 × 1	GRAPPA: 2
Diffusion-weighted							
b = 1000	Twice-refocused SE	9100	104		192 × 192	2 × 2 × 2	directions: 30; slices: 66 (axial); averages: 1
b = 2000	Twice-refocused SE	9100	104		192 × 192	2 × 2 × 2	directions: 30; slices: 66 (axial); averages: 1
b = 0	Twice-refocused SE	9100	104		192 × 192	2 × 2 × 2	slices: 66 (axial);images: 3
Magnetisation transfer							
Baseline	MT-prepared SPGR	30^a^	5		192 × 192	1.5 × 1.5 × 1.5	bandwidth: 190 Hz/px
MT	MT-prepared SPGR	30^a^	5		192 × 192	1.5 × 1.5 × 1.5	bandwidth: 190 Hz/px; RF pulse applied^b^

Notes: TR = repetition time; TE = echo time; TI = inversion time; FOV = field of view; MPRAGE = magnetisation prepared gradient echo; SPACE = spatially-selective single-slab 3D turbo-spin-echo (Mugler and Brookeman, 2004); SE = spin echo; MT = magnetisation transfer; SPGR = spoiled gradient.

a TR = 50 used if SAR exceeded limits.

b RF pulse: Gaussian RF pulse, 1950Hz (bandwidth = 375Hz, flip angle = 500°, duration = 9984 μs).

**Table 2:** Functional MRI scans collected in Stage 2.

Scan type	Sequence	TR (ms)	TE (ms)	Flip angle (°)	FOV (mm)	Voxel Size (mm)	Volumes (N)	Slices (N)	Slice thickness (mm)	Gap (%)	Order	Task^a^
Resting state	EPI	1970	30	78	192 × 192	3 × 3 × 4.44	261	32	3.7	20	Descending	Rest with eyes closed
Movie watching	multi-echo EPI	2470	5 echoes^b^	78	192 × 192	3 × 3 × 4.44	5x 193	32	3.7	20	Descending	Watch and listen to movie
Sensori-motor task	EPI	1970	30	78	192 × 192	3 × 3 × 4.44	261	32	3.7	20	Descending	Audio-visual stimuli and manual response
Field map												
Magnitude	PE-GRE	400	2 echoes^c^	60	192 × 192	3 × 3 × 4.44	1	32	3.7	20	Descending	None
Phase	PE-GRE	400	2 echoes^c^	60	192 × 192	3 × 3 × 4.44	1	32	3.7	20	Descending	None

Notes. TR = repetition time; TE = echo time; TI = inversion time; FOV = field of view; EPI = T2*-weighted gradient echo echo planar image; PE-GRE = phase-encoded gradient echo.

a Task: see text for details.

b Multi-echo EPI TE: 9.4, 21.2, 33, 45, 57.

c PE-GRE TE: 5.19, 7.65.

**Table 3:** MEG data collected in Stage 2.

Recording type	Sampling rate (Hz)	Duration (min:s)	Task^a^
Resting state	1000	08:40	Rest with eyes closed
Sensorimotor task	1000	08:40	Audio-visual stimuli and manual response
Audio-visual task	1000	02:00	Separate auditory and visual stimuli, no manual response

a Task: See text for details.

**Table 4:** Cognitive behavioural tasks used in Stage 2.

Task name	Brief description	Key variables	References
Emotion expression recognition	View face and label emotion expressed (happy, sad, anger, fear, disgust, surprise) where faces are morphs along axes between emotional expressions.	Acc, RT for each emotion	Calder et al., 1996, Ekman and Friesen, 1976
Emotional memory	Study: View (positive, neutral, or negative) background image, then object image superimposed, and imagine a ‘story’ linking the two; Test (incidental): View and identify degraded image of (studied, new) object, then judge memory and confidence for visually intact image of same object, then recall valence and any details of background image from study phase.	For each valence: Priming (Acc for studied vs. new degraded objects); familiarity (Acc for item memory); recollection (Acc for background memory)	Mitchell, 1989, Fleischman, 2007, La Voie and Light, 1994, Lang et al., 1988
Emotional reactivity and regulation	View (positive, neutral, negative) film clips under instructions to simply ‘watch’ or ‘reappraise’ (attempt to reduce emotional impact by reinterpreting its meaning; for some negative films only), then rate emotional impact (how negative, positive they felt during clip) and the degree to which they successfully reappraised.	Reactivity (ratings for ‘watch’ trials: positive vs. neutral; negative vs. neutral); regulation (ratings for ‘reappraise’ negative vs. ‘watch’ negative)	Dalgleish, 2004, Mather, 2012, Mather and Carstensen, 2005
Face recognition: familiar faces	View faces of famous people (and some unknown foils), judge whether each is familiar, and if so, what is known about the person (occupation, nationality, origin of fame, etc.), then attempt to provide person's name.	Acc (identifying information or full name given) as a proportion of number of faces recognised as familiar, subtracting false alarms (unknown faces given ‘familiar’ response)	Germine and Hooker, 2011, Bartlett and Leslie, 1986
Face recognition: unfamiliar faces	Given a target image of a face, identify same individual in an array of 6 face images (with possible changes in head orientation and lighting between target and same face in the test array)	Acc	Benton et al., 1983, Levin et al., 1975
Fluid intelligence	Complete nonverbal puzzles involving series completion, classification, matrices, and conditions.	Acc on each of 4 subtests	Cattell, 1971; Testing IfPaA, 1973, Kievit et al., 2014
Force matching	Match mechanical force applied to left index finger by using right index finger either directly, pressing a lever which transmits force to left index finger, or indirectly, by moving a slider which adjusts the force transmitted to the left index finger.	Average difference between target force and matched force applied by participant via (direct, indirect) means	
Hotel task	Perform tasks in role of hotel manager: write customer bills, sort money, proofread advert, sort playing cards, alphabetise list of names. Total time must be allocated equally between tasks; there is not enough time to complete any one task.	Number of tasks attempted, deviation from optimal time allocation	Shallice and Burgess, 1991, Kievit et al., 2014
Motor learning	Time-pressured movement of a cursor to a target by moving an (occluded) stylus under veridical, perturbed (30°), and reset (veridical again) mappings between visual and real space.	RT (movement time to hit target), trajectory error (angle) across phases	
Picture-picture priming	Name the pictured object presented alone (baseline), then when preceded by a prime object that is phonologically related (one, two initial phonemes), semantically related (low, high relatedness), or unrelated.	Acc, RT, priming effects (RT of each condition vs. baseline)	
Proverb comprehension	Read and interpret three English proverbs.	Sum of response ratings (1 = incorrect or “don't know”, 2 = partly correct but literal, 3 = correct and abstract)	Hodges, 1994
Sentence comprehension	Listen to and judge grammatical acceptability of partial sentences, beginning with an (ambiguous, unambiguous) sentence stem (e.g., “Tom noticed that landing planes…”) followed by a disambiguating continuation word (e.g., “are”) in a different voice. Ambiguity is either semantic or syntactic, with empirically determined dominant and subordinate interpretations.	RT, proportion of “unacceptable” responses in each condition	Rodd et al., 2010, Tyler et al., 2011
Tip-of-the-tongue task	View faces of famous people (actors, musicians, politicians, etc.) and respond with the person's name, or “don't know” if they do not know the person's name (even if familiar), or “TOT” if they know the person's name but are (temporarily) unable to retrieve it.	Proportion of responses of each type; incorrect “Know” responses; partial information responses (e.g., occupation)	Lovelace and Twohig, 1990, Reese et al., 1999
Visual short-term memory	View (1–4) coloured discs briefly presented on a computer screen, then after a delay, attempt to remember the colour of the disc that was at a cued location, with response indicated by selecting the colour on a colour wheel (touchscreen input).	Parameters of model fitted to error distribution: VSTM capacity (k), precision, probability of reporting an un-cued item	Zhang and Luck, 2008

Notes. Acc = accuracy; RT = response times.

## Footnotes

1 In the data repository, Stages 1, 2, and 3 are referred to as CC3000, CC700, and CC280, respectively.
